# Clinical Course and Risk Factors of Lenvatinib‐Related Hypothyroidism in Hepatocellular Carcinoma: A Retrospective Cohort Study

**DOI:** 10.1002/cnr2.70461

**Published:** 2026-01-20

**Authors:** Chihiro Shiraishi, Hideo Kato, Yuki Asai, Takuya Iwamoto

**Affiliations:** ^1^ Department of Pharmaceutical Sciences for Health Crisis Management, Faculty of Pharmaceutical Sciences Fukuoka University Fukuoka Japan; ^2^ Department of Pharmacy Mie University Hospital Mie Japan; ^3^ Department of Clinical Pharmaceutics, Division of Clinical Medical Science Mie University Graduate School of Medicine Mie Japan

**Keywords:** lenvatinib‐related hypothyroidism, thyroid dysfunction, tyrosine kinase inhibitor

## Abstract

**Background:**

Lenvatinib‐related hypothyroidism occurs in 16%–22% of patients with hepatocellular carcinoma and may affect treatment management. Previous studies have reported elevated serum thyroid stimulating hormone (TSH) levels despite levothyroxine therapy, indicating the need for research on the optimal levothyroxine dose during lenvatinib therapy.

**Aims:**

We aimed to explore the effect of lenvatinib on thyroid function, focusing on lenvatinib pharmacokinetics and factors influencing hypothyroidism development.

**Methods and Results:**

A retrospective cohort study involving 56 patients with hepatocellular carcinoma treated with lenvatinib was conducted at the Mie University Hospital. The primary outcome was grade ≥ 2 hypothyroidism. Demographic data, treatment details, and clinical outcomes were analyzed to identify factors associated with lenvatinib‐related hypothyroidism. In addition, we assessed the levothyroxine dose administered during treatment and the dose required to achieve TSH levels < 10 mIU/L. Twelve patients (21%) developed hypothyroidism during lenvatinib therapy. The median levothyroxine dose and time to onset of hypothyroidism were 25.0 [25.0–50.0] μg/day and 81.0 [15.0–208.0] days, respectively. Levothyroxine therapy was initiated at either a low or recommended dose, with 10 patients achieving TSH levels < 10 mIU/L. Significant predictors of hypothyroidism included age ≤ 63 years and a higher daily lenvatinib dose (≥ 0.152 mg/kg/day).

**Conclusion:**

Initiation of levothyroxine therapy, even at lower doses, may be effective in managing lenvatinib‐related hypothyroidism. Underweight patients (e.g., body weight < 50 kg) require particular attention due to their increased susceptibility to hypothyroidism. Even though this study has limitations such as a small sample size and the lack of multivariate analysis, it provides valuable information for managing lenvatinib‐related hypothyroidism.

**Abbreviations**
Alb: serum albumin
ALP: alkaline phosphatase
ALT: alanine aminotransferase
AST: aspartate aminotransferase
AUC: area under the receiver operating characteristic curve
BMI: body mass index
BUN: blood urea nitrogen
CHD: coronary heart disease
CI: confidence interval
CPK: creatine phosphokinase
eGFR: estimated glomerular filtration rate
FIB‐4: fibrosis‐4
fT3: free triiodothyronine
fT4: free thyroxine
ICI: immune checkpoint inhibitor
LT4: levothyroxine
OR: odds ratio
SCr: serum creatinine
T‐Bil: total bilirubin
T‐Chol: total cholesterol
TKI: tyrosine kinase inhibitor
TPO: thyroid peroxidase
TSH: thyroid‐stimulating hormone

## 1 Introduction

Lenvatinib is an oral tyrosine kinase inhibitor (TKI) that targets VEGFR 1–3, fibroblast growth factor receptors 1–4, platelet‐derived growth factor receptor alpha, rearranged during transfection, and stem cell factor receptor oncogenes [1, 2, 3, 4]. Up to 52% of patients with hepatocellular carcinoma develop overt hypothyroidism during lenvatinib treatment [5]. Given that the symptoms and signs of hypothyroidism are neither sensitive nor specific, periodic assessment of thyroid‐stimulating hormone (TSH) levels is used to monitor the adequacy of levothyroxine therapy [6].

Levothyroxine replacement therapy is initiated in patients with symptomatic hypothyroidism or TSH > 10 mIU/L [7]. The recommended levothyroxine dose for adults is 1.0–1.6 μg/kg/day, while a lower initial dose of 12.5–25.0 μg (approximately 0.6 μg/kg/day) is recommended for older patients and those with coronary artery disease [8, 9]. In general, levothyroxine dose adjustments are made based on TSH values every 4–6 weeks until target levels are achieved [8]. However, it remains unclear to what extent the effects of lenvatinib on thyroid hormone metabolism—such as alterations in thyroid vasculature, antibody production, enzyme activity, and iodine uptake—may impact the required dosage of levothyroxine [10, 11, 12]. In fact, persistent TSH elevation despite levothyroxine treatment has been reported in patients with lenvatinib‐related hypothyroidism [8].

To date, only a few studies have identified factors associated with high‐grade lenvatinib‐related hypothyroidism [5, 13], and no predictive equations or consensus guidelines are available to inform optimal levothyroxine dosing during lenvatinib therapy, highlighting a significant gap in current knowledge. Therefore, further research is needed to determine the optimal dose of levothyroxine required to reduce TSH levels in patients with lenvatinib‐related hypothyroidism. The subclinical thyroid dysfunction, characterized by TSH levels outside the reference range but fT4 within the normal range, is also associated with various adverse clinical outcomes, including an increased risk of coronary heart disease (CHD) and cardiovascular mortality, particularly at extreme TSH levels [14, 15].

Hence, this study aimed to evaluate the factors contributing to lenvatinib‐related hypothyroidism, the levothyroxine dosage during lenvatinib treatment, and the required dose to achieve TSH levels < 10 mIU/L.

## 2 Methods

### 2.1 Patient Population

We conducted a retrospective cohort study of patients treated with lenvatinib for hepatocellular carcinoma at Mie University Hospital from March 2018 to January 2024. Patients were excluded if they met any of the following criteria: a history of hypothyroidism, a single TSH measurement, no baseline TSH measurement, and initiation of levothyroxine treatment at another hospital where details could not be traced. In a certain study to evaluate the impact of prior immune checkpoint inhibitor (ICI) therapy on the development of hypothyroidism during lenvatinib treatment, patients with a history of ICI therapy were included.

### 2.2 Data Collection

We collected demographic data (sex, age, body weight, body mass index, anti‐thyroglobulin antibody, and antithyroid peroxidase antibody), details of lenvatinib and levothyroxine treatment (daily dose and treatment duration), clinical laboratory data (TSH, free thyroxine [fT4], free triiodothyronine [fT3], serum albumin, alkaline phosphatase, alanine aminotransferase [ALT], aspartate aminotransferase [AST], total bilirubin, blood urea nitrogen, serum creatinine [SCr], estimated glomerular filtration rate [eGFR], fibrosis‐4 index, total bilirubin, total cholesterol, and creatine phosphokinase [CPK]), and medical records (history of heart disease and treatment with ICIs). Lenvatinib dosing was classified based on the package insert for hepatocellular carcinoma: 12 mg/day for patients ≥ 60 kg and 8 mg/day for those < 60 kg. Dosing was further categorized as underdosed or approved [16]. Furthermore, we used the weight‐adjusted dose (mg/kg/day) as a surrogate marker to investigate whether being underweight or overweight influences the development of hypothyroidism. We also collected data on concomitant medications affecting the pharmacokinetics and pharmacodynamics of levothyroxine (steroids, proton pump inhibitors, phosphate binders, and zinc preparation) [17], and symptoms of hypothyroidism (lethargy, fatigue, eyelid edema, cold intolerance, weight gain, slow movements, poor memory, constipation, and hoarseness) were identified based on medical records provided by the medical staff. Since the initial lenvatinib dose varied throughout the study period, the relative dose intensity (RDI) was calculated as the ratio of the actual delivered dose intensity to the standard dose intensity during lenvatinib therapy [18]. The fibrosis‐4 index was determined using the following formula [19]:

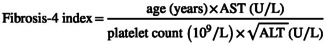




The eGFR was calculated using the prediction equation, validated for Japanese patients [20]:

$$\begin{array}{cc} \text{eGFR}\;\left(\mathrm{mL}/\min/1.73\;m^{2}\right)=\; & 194\times {\mathrm{SCr}}^{-1.094}\\ & \times {\mathrm{age}}^{-0.287}\;\left(\times 0.739\;\text{if female}\right) \end{array}$$

Based on the increased risk of CHD in subclinical hypothyroidism, current guidelines recommend levothyroxine treatment for TSH levels > 10 mIU/L regardless of fT4 values [21, 22]. Data were collected between the initiation and discontinuation of lenvatinib therapy. We investigated the date on which TSH level > 10 mIU/L, the initiation date of levothyroxine therapy, the dose administered during lenvatinib treatment, and the levothyroxine amount required to reduce TSH level to < 10 mIU/L. For patients with symptoms suggestive of hypothyroidism (lethargy, fatigue, eyelid edema, cold intolerance, weight gain, slow movements, poor memory, constipation, and hoarseness), clinical improvement following levothyroxine initiation was also evaluated. Levothyroxine dose was categorized based on patient age and the presence of heart disease. For patients aged < 65 years without heart disease, the standard adult recommended dose of 1.0–1.6 μg/kg/day was applied. Doses were classified as follows: < 1.0 μg/kg/day = “Below the recommended dose,” 1.0–1.6 μg/kg/day = “Recommended,” > 1.6 μg/kg/day = “Above the recommended dose.” For patients aged ≥ 65 years or with heart disease, a lower initial dose (approximately ≤ 0.6 μg/kg/day) was considered appropriate. In this group, doses ≤ 0.6 μg/kg/day were classified as “Recommended,” and those > 0.6 μg/kg/day as “Above the recommended dose” [8, 9].

### 2.3 Statistical Analyses

Statistical analyses were performed using the JMP Pro 16 statistical package (SAS Institute, Cary, NC, USA). Categorical data were summarized as counts (%) and analyzed using the chi‐square test. Continuous data were summarized as medians (interquartile range) and analyzed using the Mann–Whitney *U* test. A two‐tailed *p* value of < 0.05 was considered statistically significant. Given the limited sample size and number of events, we performed univariable analyses to explore associations between candidate factors and lenvatinib‐related hypothyroidism. Multivariable analysis was not planned, and the univariable findings should be interpreted as exploratory rather than confirmatory. The cut‐off values for continuous variables predicting lenvatinib‐related hypothyroidism were determined using receiver operating characteristic (ROC) curve analysis. Missing values were handled without imputation. Because of the single‐center study design and the incidence rate of hypothyroidism with lenvatinib observed in previous studies, multivariable analysis was not planned in the study protocol.

### 2.4 Ethics Approval

This study was conducted in accordance with the Declaration of Helsinki and its amendments, following approval from the Clinical Research Ethics Review Committee of Mie University Hospital (No. H2023‐034). Data were accessed for research purposes after February 27, 2024. Informed consent was obtained from all participants through an opt‐out method, as the data were retrospectively collected from electronic medical records. Information that could identify individual participants was accessed only after data collection. Data were collected during lenvatinib therapy.

## 3 Results

### 3.1 Baseline Characteristics

**FIGURE 1:**
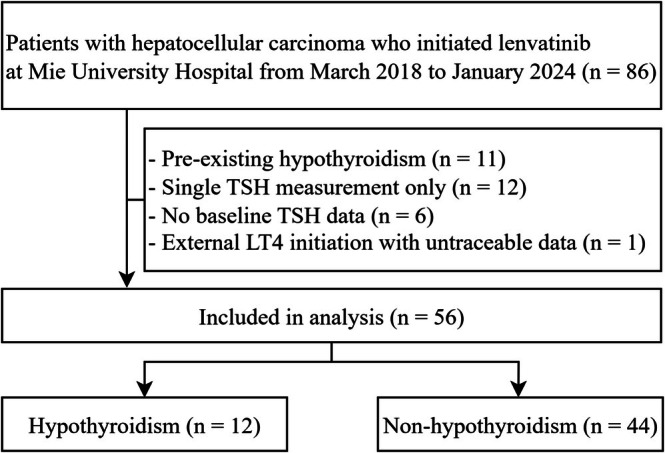
Flowchart of the patient selection process. LT4, levothyroxine; TSH, thyroid‐stimulating hormone.

During the study period, lenvatinib was administered to 86 patients. Figure 1 presents a flowchart of the patient selection process. The exclusion criteria were as follows: patients with a history of hypothyroidism (*n* = 11), only a single TSH measurement (*n* = 12), absence of baseline TSH measurement (*n* = 6), and case in which levothyroxine was initiated at other hospitals with untraceable details (*n* = 1). A total of 56 patients were enrolled in this study and 12 (21%) developed hypothyroidism during lenvatinib therapy.

The baseline characteristics of the patients are summarized in Table 1. The median age, body weight, and daily lenvatinib dose per body weight were 73.0 [66.0–79.0] years, 61.0 [52.9–68.5] kg, and 0.151 [0.124–0.171] mg/kg, respectively. Ten (18%) patients had a history of ICI treatment. Patients who received previous ICI therapy had no other endocrinopathies. Seventeen (30%) patients received lenvatinib at an underdose, while 39 (70%) received the approved dose. One (2%) patient had anti‐thyroglobulin antibodies, and seven (13%) had anti‐TPO antibodies. Among the concomitant medications, 30 (54%) received proton pump inhibitors. Among the 44 patients without hypothyroidism, 18 (41%) initiated lenvatinib at a lower dose based on their clinical condition. There were no patients with Child‐Pugh class C liver disease or co‐administration of medications known to significantly interact with lenvatinib.

**TABLE 1:** Patients baseline characteristics.

	*n* (%)
Female	13 (23)
Age (year)	73.0 [66.0–79.0]
Body weight (kg)	61.0 [52.9–68.5]
BMI (kg/m^2^)	23.2 [21.1–25.4]
Heart disease	12 (21)
Anti‐thyroglobulin antibody	1 (2)
Anti TPO antibody	7 (13)
Daily lenvatinib dose	
4 mg/day	5 (9)
8 mg/day	35 (63)
12 mg/day	16 (29)
Adequate dose	39 (70)
Underdose	17 (30)
Daily lenvatinib dose per body weight, mg/kg	0.151 [0.124–0.171]
RDI	71.0 [52.2–91.1]
TSH (mIU/L)	1.9 [1.1–2.7]
fT4 (ng/dL)	1.0 [0.9–1.1]
fT3 (pg/dL)	2.5 [2.2–2.7]
Alb (g/dL)	3.8 [3.3–4.2]
ALP (U/L)	343.0 [252.7–607.5]
ALT (U/L)	38.0 [21.0–53.0]
AST (U/L)	44.0 [31.0–65.0]
BUN (mg/dL)	14.9 [11.5–20.8]
SCr (mg/dL)	0.8 [0.6–1.0]
eGFR (mL/min/1.73 m^2^)	70.3 [53.4–86.8]
FIB‐4 index	3.7 [2.1–6.0]
T‐Bil (mg/dL)	0.9 [0.6–1.2]
T‐Chol (mg/dL)	166.5 [145.0–201.0]
CPK (U/L)	94.5 [62.0–150.0]
Treatment history of immune checkpoint inhibition	10 (18)
Concomitant medications	
Steroid	3 (5)
Proton pump inhibitor	30 (54)
Phosphate adsorbent	1 (2)
Zinc preparation	3 (5)

*Note:* eGFR (mL/min/1.73 m^2^) = 194 × SCr^−1.094^ × age^−0.287^ (× 0.739 if female) [20].

Data are presented as median [interquartile range].

Abbreviations: Alb, serum albumin; ALP, alkaline phosphatase; ALT, alanine aminotransferase; AST, aspartate aminotransferase; BMI, body mass index; BUN, blood urea nitrogen; CPK, creatine phosphokinase; eGFR, estimated glomerular filtration rate; FIB‐4, fibrosis‐4; fT3, free triiodothyronine; fT4, free thyroxine; RDI, relative dose intensity; SCr, serum creatinine; T‐Bil, total bilirubin; T‐Chol, total cholesterol; TPO, thyroid peroxidase; TSH, thyroid‐stimulating hormone.

Table 2A presents the baseline characteristics of the 12 patients who developed hypothyroidism, and Table 2B summarizes their clinical course after levothyroxine initiation, including dose adjustments. In these patients, the median levothyroxine dose, levothyroxine dose per body weight, and onset date of hypothyroidism were 25.0 [25.0–50.0], 0.5 [0.3–1.3], and 81.0 [15.0–208.0] days, respectively. Levothyroxine therapy was initiated at a lower or recommended dose in 11/12 patients, and 10 patients achieved TSH levels < 10 mIU/L, with a median of 49 [35–127] days following levothyroxine initiation. Of the four patients who presented with hoarseness or fatigue, two experienced symptom improvement within the recommended TSH range. There were no significant differences in RDI between patients with and without hypothyroidism (hypothyroidism, 78.1 [53.7–91.0] vs. non‐hypothyroidism, 70.3 [49.3–92.9]; *p* = 0.631) (Table S1).

**TABLE 2A:** Baseline characteristics and initial clinical course before levothyroxine initiation.

No.	Age (years)	TSH (mIU/L)	fT4 (ng/dL)	Heart disease	Day of first TSH > 10 mIU/L	Symptoms (onset)	Days from first TSH > 10 mIU/L to LT4 initiation	LT4 dose (μg/day)	LT4 dose (μg/kg/day)	Dose category
1	68	1.9	0.8	No	89	Hoarseness (35)	141	25	0.4	Recommended
2	53	2.1	1.2	No	21	—	12	50	1.3	Recommended
3	62	3.8	0.9	No	17	Hoarseness (35) Fatigue (35)	165	25	0.6	Below the recommended dose
4	78	2.3	1.0	No	115	—	155	25	0.5	Recommended
5	76	2.9	0.9	No	87	—	57	25	0.4	Recommended
6	61	1.8	1.3	No	208	Fatigue (19) Hoarseness (334)	0	25	0.5	Below the recommended dose
7	82	1.3	1.0	No	NA	—	NA	50	1.3	Above the recommended dose
8	74	2.4	1.1	Yes	200	—	165	25	0.4	Recommended
9	34	4.8	1.1	No	22	—	424	25	0.6	Below the recommended dose
10	60	3.9	0.9	No	40	—	41	25	0.5	Below the recommended dose
11	59	0.6	0.9	No	14	—	29	25	0.6	Below the recommended dose
12	52	1.0	1.1	No	93	Hoarseness (43)	76	25	0.3	Below the recommended dose

*Note:* Day indicates days from lenvatinib initiation. The recommended levothyroxine dose for adults is 1.0–1.6 μg/kg/day, while a lower initial dose of 12.5–25.0 μg (approximately 0.6 μg/kg/day) is recommended for older patients and those with coronary artery disease [8, 9]. Levothyroxine dose was categorized based on patient age and the presence of heart disease. For patients aged < 65 years without heart disease, the standard adult recommended dose of 1.0–1.6 μg/kg/day was applied. Doses were classified as follows: < 1.0 μg/kg/day = “Below the recommended dose,” 1.0–1.6 μg/kg/day = “Recommended,” > 1.6 μg/kg/day = “Above the recommended dose.” For patients aged ≥ 65 years or with heart disease, a lower initial dose (approximately ≤ 0.6 μg/kg/day) was considered appropriate. In this group, doses ≤ 0.6 μg/kg/day were classified as “Recommended,” and those > 0.6 μg/kg/day as “Above the recommended dose” [8, 9]. NA indicates that TSH did not exceed 10 mIU/L prior to LT4 initiation; therefore, the interval could not be calculated. 0 indicates LT4 was initiated on the same day as the first TSH > 10 mIU/L measurement. A dash (—) indicates no symptoms.

Abbreviations: fT4, free thyroxine; LT4, levothyroxine; TSH, thyroid‐stimulating hormone.

**TABLE 2B:** Response to levothyroxine therapy and clinical course after initiation.

No	TSH at LT4 initiation (mIU/L)	fT4 at LT4 initiation (ng/dL)	TSH after LT4 initiation (mIU/L)	Symptom response after LT4 initiation	Dose adjusted (new LT4 dose, μg/kg/day)	TSH after dose adjustment (mIU/L)	Symptom response after dose adjustment
1	15.0	0.8	3.9	Improvement	No	—	—
2	38.6	0.6	0.3	NA	No	—	—
3	14.7	0.9	7.1	No improvement	Yes (0.8)	0.5	Improvement
4	41.4	0.7	21.8	NA	Yes (1.0)	16.7	NA
5	28.8	0.9	7.3	NA	No	—	—
6	9.1	1.0	3.9	No improvement	Yes (1.0)	1.0	Improvement
7	6.5	1.0	5.8	NA	No	—	—
8	15.8	1.0	NA	NA	Yes (0.7)	1.4	NA
9	213.6	0.8	91.1	NA	No (death before dose adjustment)	—	—
10	23.7	0.8	8.8	NA	No	—	—
11	36.4	0.9	4.5	NA	No	—	—
12	96.2	0.7	5.6	Improvement	No	—	—

*Note:* TSH after LT4 initiation refers to the first follow‐up TSH measurement after LT4 initiation (before dose adjustment, when applicable). Symptom response was assessed only in patients with symptoms suggestive of hypothyroidism.

Abbreviations: —, not applicable; fT4, free thyroxine; LT4, levothyroxine; NA, not assessed or not available; TSH, thyroid‐stimulating hormone.

### 3.2 Univariate Logistic Regression Analysis of Lenvatinib‐Related Hypothyroidism

**FIGURE 2:**
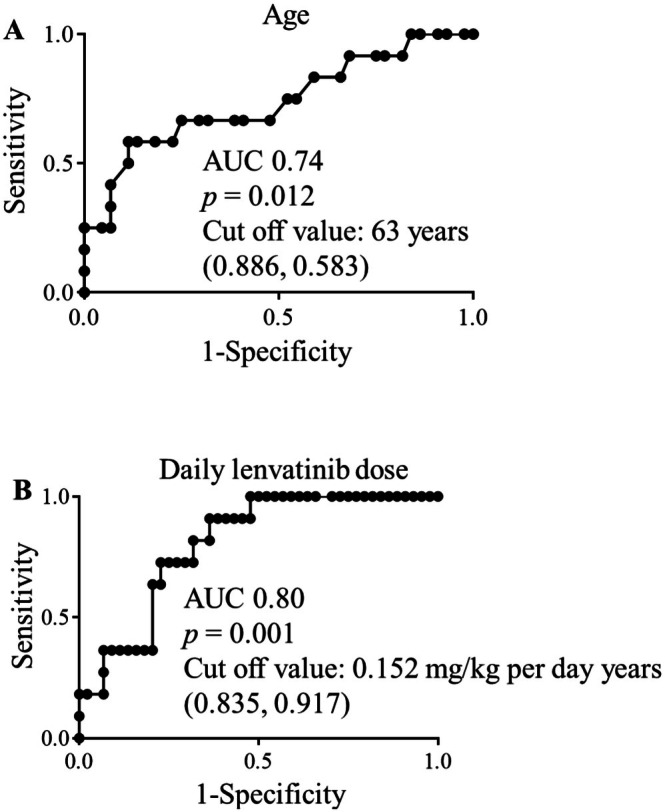
(A) Cut‐off value and the AUC corresponding to the results of the ROC analysis for age. The *X*‐axis represents specificity, and the *Y*‐axis represents sensitivity. The cutoff value was 63 years, AUC = 0.74, *p* = 0.012 (specificity, 0.583; sensitivity, 0.886). (B) Cut‐off value and AUC corresponding to the results of the ROC analysis for the daily lenvatinib dose per body weight. The *X*‐axis represents specificity and the *Y*‐axis represents sensitivity. The cutoff value was 0.152 mg/kg/day (AUC = 0.80, *p* = 0.001; specificity, 0.917; sensitivity, 0.835). AUC, area under the receiver operating characteristic curve; ROC, receiver operating characteristic.

Age (0.905 [0.841–0.975], *p* = 0.003) and daily lenvatinib dose per body weight (1.569 [1.148–2.220], *p* < 0.001) were associated with lenvatinib‐related hypothyroidism in univariable analyses (Table 3). The cutoff values and the area under the receiver operating characteristic curve (AUC) corresponding to the results of the ROC analysis were as follows: age, 63 years, AUC = 0.74, *p* = 0.012 (specificity, 0.583; sensitivity, 0.886) (Figure 2A); daily lenvatinib dose per body weight, 0.152 mg/kg/day, AUC = 0.80, *p* = 0.001 (specificity, 0.917; sensitivity, 0.835) (Figure 2B).

**TABLE 3:** Univariable logistic regression analysis.

	OR [95% CI]	*p*
Female	0.514 [0.126–2.098]	0.363
Age	0.905 [0.841–0.975]	0.003
Body weight	0.951 [0.893–1.015]	0.123
BMI	0.848 [0.687–1.047]	0.113
Heart disease	0.273 [0.032–2.360]	0.177
Daily lenvatinib dose	1.243 [0.929–1.663]	0.133
Daily lenvatinib dose per body weight	1.569 [1.148–2.220]	< 0.001
TSH	1.232 [0.743–2.044]	0.066
fT4	1.416 [0.060–33.458]	0.090
fT3	1.103 [0.139–7.186]	0.919
Alb	3.526 [0.653–19.036]	0.129
ALP	1.000 [0.998–1.002]	0.901
ALT	1.001 [0.977–1.026]	0.926
AST	0.999 [0.982–1.015]	0.869
BUN	0.995 [0.946–1.046]	0.830
SCr	0.897 [0.505–1.594]	0.640
eGFR	0.993 [0.965–1.022]	0.647
eGFRcys	1.048 [0.956–1.149]	0.256
FIB‐4 index	0.632 [0.254–1.129]	0.042
T‐Bil	0.603 [0.142–2.561]	0.469
T‐Chol	1.000 [0.971–1.030]	0.996
CPK	1.005 [0.992–1.018]	0.496
Steroid	1.909 [0.158–23.039]	0.624
Proton pump inhibitor	3.286 [0.783–13.789]	0.086

*Note:* eGFR (mL/min/1.73 m^2^) = 194 × SCr^−1.094^ × age^−0.287^ (× 0.739 if female) [20].

Abbreviations: Alb, serum albumin; ALP, alkaline phosphatase; ALT, alanine aminotransferase; AST, aspartate aminotransferase; AUC, area under the receiver operating characteristic curve; BMI, body mass index; BUN, blood urea nitrogen; CI, confidence interval; CPK, creatine phosphokinase; eGFR, estimated glomerular filtration rate; FIB‐4, fibrosis‐4; fT3, free triiodothyronine; fT4, free thyroxine; OR, odds ratio; SCr, serum creatinine; T‐Bil, total bilirubin; T‐Chol, Total Cholesterol; TPO, thyroid peroxidase; TSH, thyroid‐stimulating hormone.

## 4 Discussion

This study suggests that patients aged ≤ 63 years and those receiving ≥ 0.152 mg/kg/day of lenvatinib may identify a high‐risk group for hypothyroidism and therefore warrant closer monitoring of thyroid function and earlier consideration of levothyroxine replacement. Underweight patients receiving lenvatinib for hepatocellular carcinoma are also at increased risk of hypothyroidism, likely due to higher relative drug exposure per kilogram of body weight. This is biologically plausible given that lenvatinib is administered at a largely fixed daily dose; patients with lower body weight are consequently exposed to a higher dose per kilogram. Notably, low‐dose levothyroxine therapy appeared effective, with many patients achieving normalization of TSH levels and resolution of symptoms at doses below standard recommendations.

Several studies have reported that patients with TKI‐related hypothyroidism experience significantly higher oncologic response rates, progression‐free survival, and overall survival compared to those with normal thyroid function [23, 24]. Side effects associated with lenvatinib, such as decreased appetite, fatigue, and voice disorders, overlap with the symptoms of hypothyroidism, complicating the identification of thyroid dysfunction based solely on clinical presentation. Regardless of thyroid status, circulating TSH levels are positively correlated with apolipoprotein B‐containing lipoprotein cholesterols levels [25, 26, 27, 28, 29, 30]. Hypothyroidism can contribute to lipid metabolism disorders [31], primarily by increasing total cholesterol and low‐density lipoprotein cholesterol levels [32]. Additionally, hypothyroidism often causes weakness, cramps, myalgia, and mild‐to‐moderate elevation of serum CPK [33, 34, 35]. However, the absence of consistent fluctuations in laboratory data, such as increased cholesterol or CPK levels, suggests that the clinical diagnosis of hypothyroidism in patients receiving lenvatinib is challenging and primarily relies on TSH assays. Therefore, a thorough evaluation of lenvatinib‐related hypothyroidism is essential for a more comprehensive approach.

Hypothyroidism occurred more frequently in patients who received an initial lenvatinib dose of ≥ 0.152 mg/kg/day (Table 3 and Figure 2). In patients with hepatocellular carcinoma, the lenvatinib dosing is typically based on body weight, with 12 mg for patients weighing ≥ 60 kg, and 8 mg for those weighing < 60 kg [16]. A clear exposure–response relationship was observed for time‐to‐first treatment‐related side effects leading to drug withdrawal or dose reduction, as evident in the Kaplan–Meier plots, and a higher lenvatinib AUC in pharmacokinetic analysis was associated with earlier dose reductions [16]. In this study, there were no significant differences in RDI between patients with and without hypothyroidism. Taking together, these data suggest that even when an apparently reduced initial dose is selected in underweight patients, the actual weight‐based dose (mg/kg/day) can still exceed the risk threshold identified by the ROC analysis, resulting in sufficient treatment intensity but an increased risk of hypothyroidism.

Factors associated with the onset of hypothyroidism included age ≤ 63 years (Table 3 and Figure 2). When the dose was reduced from 12 to 8 mg, the maximum drug concentration of lenvatinib was reduced by nearly 50% and the AUC decreased by 55% [16, 36]. Maintaining > 70% RDI with lenvatinib has been reported as an independent factor for improved therapeutic response and prolonged progression‐free survival [37]. However, in clinical settings, lenvatinib is often initiated at a lower dose based on the patient's condition, which may have influenced our results. Among the 44 patients without thyroid dysfunction, 18 (41%) initially received a reduced lenvatinib dose, whereas all patients who developed hypothyroidism received lenvatinib without dose reduction, according to the package insert (Table S1). Therefore, it may not be that younger patients are at a higher physiological risk; rather, the prevalence of hypothyroidism may have resulted from the reduced dosage of lenvatinib in the elderly patient.

Among the patients who developed hypothyroidism during lenvatinib treatment, six (50%) initiated levothyroxine therapy at a dose lower than the recommended starting dose (Table 2A). Among them, five (80%) successfully achieved TSH levels < 10 mIU/L. In two of the four cases presenting with hypothyroidism‐related symptoms (e.g., hoarseness and fatigue), symptoms improved to within the recommended TSH range (0.4–2.5 mIU/L). This suggests that both symptom improvement and TSH normalization can be achieved even with a low dose of levothyroxine. For example, the European Thyroid Association guidelines on subclinical hypothyroidism [21] distinguish between TSH levels below and above 10 mIU/L when considering levothyroxine treatment. These recommendations are based on findings from the Thyroid Studies Collaboration that demonstrated a higher relative risk of CHD with TSH levels ≥ 10 mIU/L [14]. These findings highlight the importance of early intervention for the management of hypothyroidism.

Lenvatinib is expected to exacerbate the incidence of hypothyroidism in patients who have been treated with drugs potentially associated with thyroid dysfunction. However, prior ICI therapy was not identified as a significant factor associated with the development of hypothyroidism in this study, which is inconsistent with previous findings [5]. In that study, although the sample size was small, the reported incidences of hypothyroidism were 13% in the first lenvatinib group (*n* = 39) and 46% in the second lenvatinib group after prior atezolizumab plus bevacizumab (*n* = 13). The higher incidence observed in the second group may be influenced by prior exposure to ICI [5], which has been associated with autoimmune‐mediated destructive thyroiditis, suggesting a distinct pathophysiological mechanism and vascular changes that could exacerbate thyroid injury [37]. The mechanisms underlying lenvatinib‐related hypothyroidism are believed to include direct cytotoxic effects on thyroid follicular cells, reduced thyroidal blood flow, and possibly the induction of autoimmune responses [22]. These findings differ from our results, and therefore robust conclusions require larger, well‐controlled studies with standardized thyroid monitoring. At present, the evidence supporting prior ICI therapy as an independent risk factor for hypothyroidism during lenvatinib treatment remains limited.

This study has several limitations. First, although two patients showed symptom improvement (hoarseness) after levothyroxine initiation, we could not confirm causality due to the lack of objective quality‐of‐life measures. Furthermore, symptoms suggestive of hypothyroidism were identified exclusively from medical records, which may have resulted in an underestimation of their frequency because of incomplete documentation. Future prospective studies integrating biochemical testing, patient‐reported outcomes, and clinical endpoints could provide a more comprehensive understanding of treatment effectiveness. Second, 5 of the 12 patients who developed hypothyroidism were over 65 years old, which may have contributed to the administration of a lower levothyroxine dose. Third, the association between blood concentration and hypothyroidism has not been clearly established. In this case, there was no difference in RDI between the hypothyroid and non‐hypothyroid groups; however, further detailed investigation is needed by evaluating blood concentrations. Fourth, although thyroid dysfunction is recommended to classify into euthyroidism, subclinical hypothyroidism, overt hypothyroidism, and thyrotoxicosis, we focused only on grade ≥ 2 hypothyroidism. Fifth, TSH monitoring was inconsistent; 12 patients had only a single TSH measurement, and six lacked baseline values. This may have led to misclassification or under recognition of hypothyroidism and, consequently, suboptimal supportive management. Sixth, no patients received iron, magnesium, or enzyme‐inducing drugs, so the influence of these on thyroid function could not be assessed. Finally, because multivariable adjustment was not performed, residual confounding cannot be excluded. Multicenter validation studies with larger sample sizes are required to confirm these findings.

## 5 Conclusion

This study highlights the importance of managing thyroid dysfunction in patients receiving lenvatinib to improve treatment outcomes and quality of life. Underweight patients may be at higher risk of hypothyroidism and could benefit from early levothyroxine supplementation. These findings emphasize the need for close collaboration between oncologists and endocrinologists to ensure timely intervention and uninterrupted cancer therapy. Further large‐scale studies are warranted to optimize management strategies.

## Author Contributions

Conceptualization, methodology, data curation, investigation, formal analysis, visualization, writing – original draft, C.S. Supervision, T.I. Writing – review and editing, H.K., A.Y., and T.I. All authors had full access to the data in the study and take responsibility for the integrity of the data and the accuracy of the data analysis.

## Funding

The authors have nothing to report.

## Ethics Statement

This study was conducted in accordance with the Declaration of Helsinki and its amendments, following approval from the Clinical Research Ethics Review Committee of Mie University Hospital (No. H2023‐034).

## Consent

The authors have nothing to report.

## Conflicts of Interest

The authors declare no conflicts of interest.

## Supporting information


**Table S1:** Patients baseline characteristics divided the onset of hypothyroidism.

## Data Availability

The data that support the findings of this study are available on request from the corresponding author. The data are not publicly available due to privacy or ethical restrictions.
