# Supplementary material for: Clinical Course and Risk Factors of Lenvatinib‐Related Hypothyroidism in Hepatocellular Carcinoma: A Retrospective Cohort Study
Source: Cancer Rep (Hoboken). 2026 Jan 20;9(1):e70461. doi: 10.1002/cnr2.70461 (PMC12819047; doi:10.1002/cnr2.70461)
Supplement: Supplementary file 1 — Table S1: Patients baseline characteristics divided the onset of hypothyroidism. [file CNR2-9-e70461-s001.docx]

TABLE S1. Patients baseline characteristics divided the onset of hypothyroidism.

|  | Hypothyroidism  (n = 12) | Non-hypothyroidism  (n = 44) | *p*–value |
| --- | --- | --- | --- |
|  | n (%) | |  |
| Female | 4 (33) | 9 (20) | 0.443 |
| Age, year | 61.5 [54.5–75.5] | 74.0 [68.3–79.8] | 0.012 |
| Body weight, kg | 54.4 [44.7–70.0] | 62.5 [54.3–67.7] | 0.138 |
| BMI, kg/m^2^ | 22.4 [17.6–24.8] | 23.4 [21.4–25.4] | 0.159 |
| Heart disease | 1 (8) | 11(25) | 0.212 |
| Anti-thyroglobulin antibody | 0 (0) | 1 (2) | 1.000 |
| Anti TPO antibody | 0 (0) | 7 (16) | 0.323 |
| Daily lenvatinib dose |  |  |  |
| 4 mg/day | 0 (0) | 5 (11) | 0.574 |
| 8 mg/day | 7 (67) | 28 (64) | 0.748 |
| 12 mg/day | 5 (42) | 11 (25) | 0.293 |
| Initial dose reduction  according to the package insert, yes | 0 (0) | 18 (40.9) | 0.006 |
| Daily lenvatinib dose per body weight, mg/kg | 0.171 [0.156–0.186] | 0.140 [0.121–0.161] | 0.001 |
| RDI | 78.1 [53.7–91.0] | 70.3 [49.3–92.9] | 0.631 |
| TSH, mIU/L | 2.2 [1.4–3.6] | 1.7 [1.1–2.5] | 0.135 |
| fT4, ng/dL | 1. [0.9–1.1] | 1. [0.9–1.1] | 0.855 |
| fT3, pg/dL | 2.5 [2.3–2.8] | 2.5 [2.2–2.7] | 0.933 |
| Alb, g/dL | 3.9 [3.6–4.2] | 3.5 [3.2–4.1] | 0.203 |
| ALP, U/L | 421.0 [201.0–768.0] | 340.8 [279.0–603.0] | 0.957 |
| ALT, U/L | 34.5 [22.0–60.0] | 38.0 [21.0–52.0] | 0.976 |
| AST, U/L | 39.0 [29.5–64.5] | 44.0 [32.0–65.0] | 0.760 |
| BUN, mg/dL | 16.3 [14.5–21.5] | 14.8 [11.3–20.1] | 0.237 |
| SCr, mg/dL | 0.9 [0.6–1.0] | 0.8 [0.6–1.0] | 0.514 |
| eGFR, ml/min/1.73m^2^ | 67.0 [51.6–81.1] | 72.3 [56.5–87.2] | 0.427 |
| FIB-4 index | 2.6 [1.4–3.9] | 4.1 [2.4–6.6] | 0.067 |
| T-Bil, mg/dL | 0.8 [0.6–1.2] | 0.9 [0.6–1.2] | 0.602 |
| T-Chol, mg/dL | 180.5 [159.3–199.5] | 158.0 [143.5–203.3] | 0.205 |
| CPK, U/L | 77.0 [60.0–156.0] | 112.0 [112.0–112.0] | 0.862 |
| Treatment history of immune checkpoint inhibition | 0 (0) | 10 (23) | 0.068 |
| **Concomitant medications** |  |  |  |
| Steroid | 1 (8) | 2 (5) | 0.522 |
| Proton pump inhibitor | 9 (75) | 21 (48) | 0.114 |
| Phosphate adsorbent | 0 (0) | 1 (2) | 1.000 |
| Zinc Preparation | 0 (0) | 3 (7) | 0.857 |

Alb, serum albumin; ALP, alkaline phosphatase; ALT, alanine aminotransferase; AST, aspartate aminotransferase; BMI, body mass index; BUN, blood urea nitrogen; CPK, creatine phosphokinase; eGFR, estimated glomerular filtration rate; FIB-4, fibrosis-4; fT3, free triiodothyronine; fT4, free thyroxine; SCr, serum creatinine; T-Bil, total bilirubin; T-Chol, Total Cholesterol; RDI, relative dose intensity; TPO, thyroid peroxidase; TSH, thyroid-stimulating hormone

eGFR (mL/min/1.73 m2) = 194 × SCr^−1.094^ × age^−0.287^ (× 0.739 if female) [20].

Data are presented as median [interquartile range].
